# Continuation of Tarlatamab With Concomitant Radiotherapy for Oligoprogression in Extensive-Stage Small-Cell Lung Cancer: A Report of Two Cases

**DOI:** 10.7759/cureus.113982

**Published:** 2026-08-05

**Authors:** Ryosuke Masui, Shota Omori, Takuya Ueno, Kosaku Komiya

**Affiliations:** 1 Department of Respiratory Medicine, National Hospital Organization (NHO) Beppu Medical Center, Beppu, JPN; 2 Department of Respiratory Medicine and Infectious Diseases, Oita University Faculty of Medicine, Yufu, JPN

**Keywords:** case report, extensive-stage small-cell lung cancer, oligoprogression, radiotherapy, tarlatamab

## Abstract

Tarlatamab, a bispecific T-cell engager immunotherapy targeting CD3 on T cells and delta-like ligand 3 on tumor cells, has demonstrated promising efficacy in patients with relapsed extensive-stage small-cell lung cancer (ES-SCLC). However, therapeutic strategies for oligoprogressive disease during tarlatamab treatment remain unestablished. We report two patients with ES-SCLC who continued tarlatamab treatment following radiotherapy for oligoprogressive disease. In Case 1, a 63-year-old man receiving fifth-line tarlatamab developed a solitary brain metastasis despite sustained extracranial disease control. Stereotactic radiotherapy achieved marked regression of the lesion, allowing continuation of tarlatamab treatment. In Case 2, a 78-year-old man receiving fourth-line tarlatamab developed isolated progression of a subcarinal lymph node metastasis after an initial partial response. Hyperfractionated radiotherapy resulted in rapid normalization of tumor marker levels and marked nodal shrinkage, enabling ongoing tarlatamab treatment. These cases suggest that radiotherapy to the progressing site may effectively control oligoprogression during tarlatamab treatment, permitting continuation of systemic therapy in selected patients with ES-SCLC. This approach may represent a useful treatment strategy for managing oligoprogressive disease during tarlatamab therapy.

## Introduction

Tarlatamab is a bispecific T-cell engager immunotherapy targeting CD3 on T cells and delta-like ligand 3 on tumor cells. In the phase two DeLLphi-301 trial involving patients with previously treated small-cell lung cancer (SCLC), tarlatamab demonstrated clinically meaningful and durable antitumor activity [1]. In the DeLLphi-304 trial, tarlatamab was compared with single-agent chemotherapy in patients with extensive-stage small-cell lung cancer (ES-SCLC) that had progressed after platinum-based chemotherapy [2]. Tarlatamab significantly improved the primary endpoint of median overall survival and the secondary endpoint of median progression-free survival (PFS) compared with chemotherapy [2]. Furthermore, the 12-month PFS rates were 20% and 4% in the tarlatamab and chemotherapy groups, respectively, suggesting that durable disease control may be achievable in a subset of patients [2]. In addition, extended follow-up of the earlier DeLLphi-300 study suggested intracranial activity in patients with brain metastases [3]. On the basis of these findings, tarlatamab has emerged as a novel treatment option for patients with relapsed ES-SCLC [1-3]. However, treatment options remain limited for patients with relapsed ES-SCLC requiring later-line therapy. Although SCLC is initially sensitive to chemotherapy, most patients relapse within six months, and effective treatment options remain limited after relapse, particularly in platinum-refractory or platinum-resistant disease [4].

Oligoprogressive disease refers to progression at a limited number of sites, while disease at other sites remains controlled during systemic therapy. When oligoprogressive disease develops during tarlatamab treatment, local therapy directed at the progressing lesions, followed by continued tarlatamab, may prolong disease control and preserve subsequent treatment options, provided that local treatment is feasible. Here, we report two patients with ES-SCLC in whom oligoprogression during tarlatamab treatment was successfully controlled with radiotherapy, allowing continuation of tarlatamab therapy.

## Case presentation

**Figure 1:**
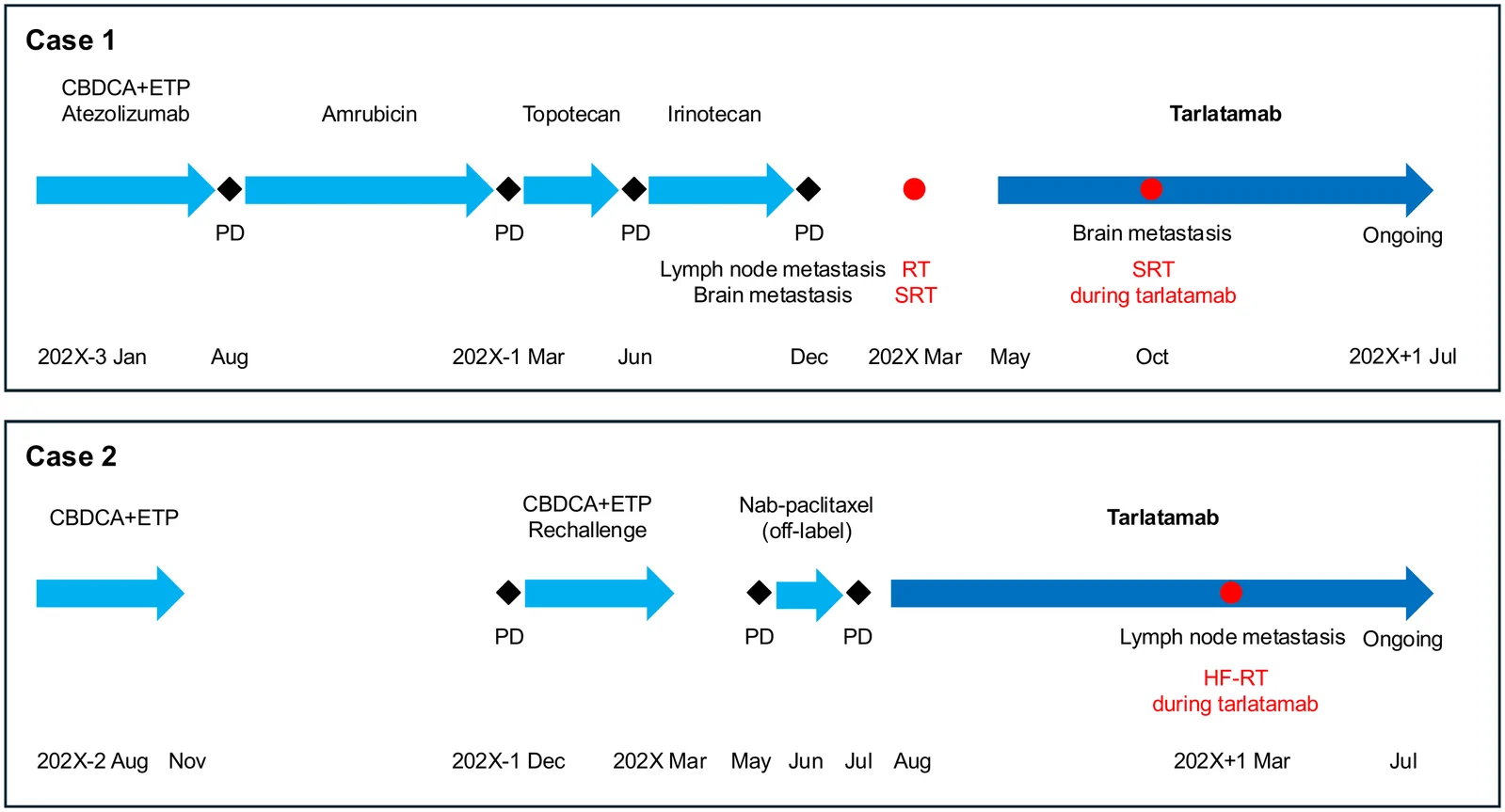
Clinical timelines of Cases 1 and 2. Oligoprogressive disease during tarlatamab treatment was managed with radiotherapy, and tarlatamab treatment was subsequently continued in both cases. CBDCA: carboplatin; ETP: etoposide; PD: progressive disease; RT: radiotherapy; SRT: stereotactic radiotherapy; HF-RT: hyperfractionated radiotherapy

This retrospective case series included two consecutive patients with ES-SCLC who received radiotherapy for oligoprogressive disease during tarlatamab treatment at the National Hospital Organization (NHO) Beppu Medical Center between May 202X and July 202X+1. Clinical information was retrospectively collected from the patients’ medical records and imaging studies. The clinical timelines of both patients are summarized in Figure 1.

Case 1

**Figure 2:**
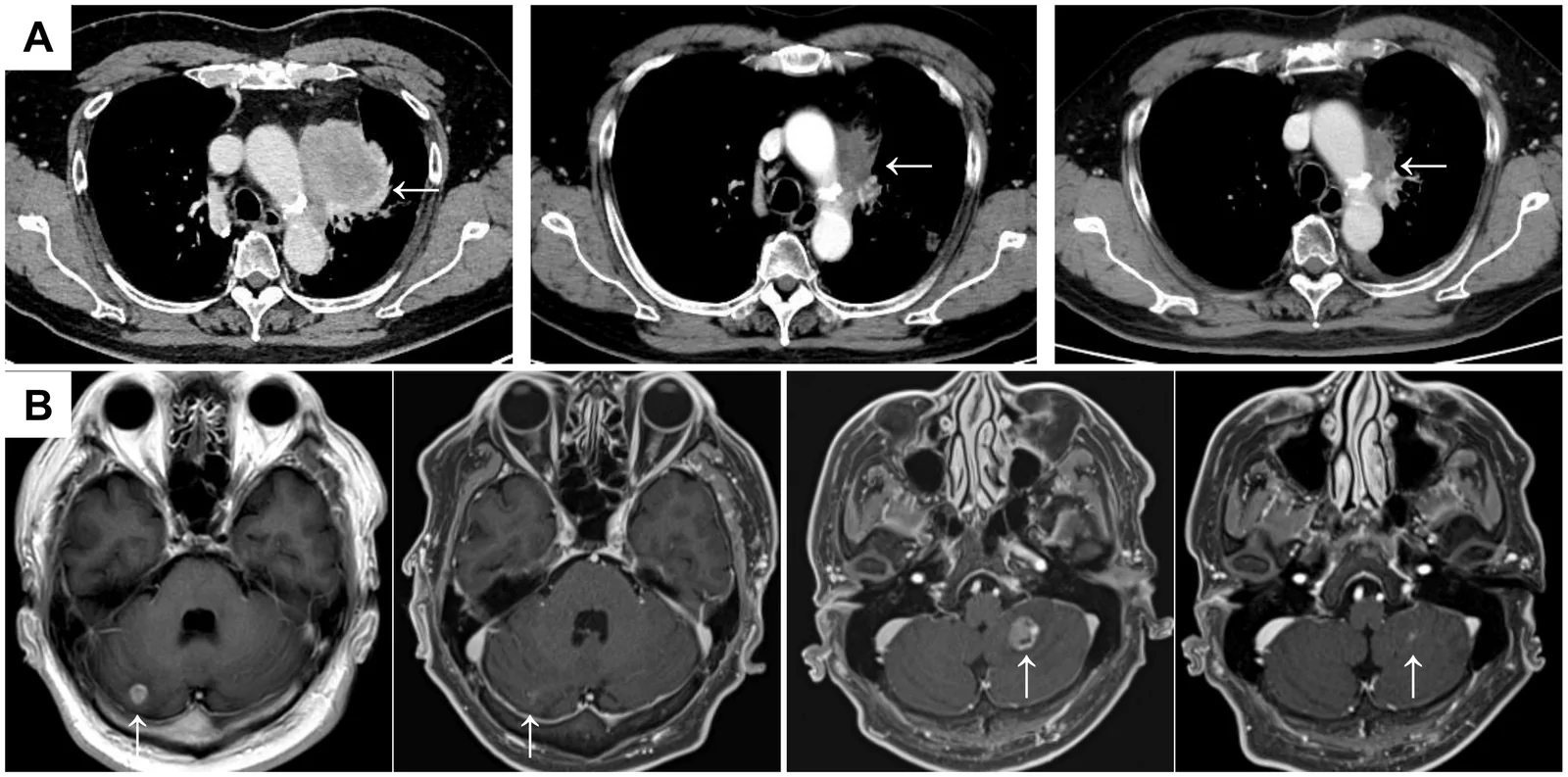
Radiological course of Case 1. (A) Computed tomography images of the para-aortic lymph node lesion before (left) and after (middle) radiotherapy. Radiotherapy was performed before tarlatamab initiation. The para-aortic lymph node lesion during tarlatamab treatment (right) shows sustained local control. (B) Magnetic resonance images showing the right cerebellar metastasis before the first course of stereotactic radiotherapy (far left) and its regression after treatment, before tarlatamab initiation (center left). A newly developed left cerebellar metastasis during tarlatamab treatment is shown before the second course of stereotactic radiotherapy (center right) and after its complete radiographic resolution following treatment (far right). White arrows indicate the para-aortic lymph node lesion and cerebellar metastases.

A 63-year-old man was referred to our hospital in December 202X-4 after an abnormal chest finding was detected during a routine health examination. SCLC was pathologically diagnosed by bronchoscopy. He had no clinically relevant comorbidities and had a 30-pack-year smoking history. His Eastern Cooperative Oncology Group (ECOG) performance status (PS) was 0 at diagnosis. He was diagnosed with ES-SCLC (cT4N2bM1a, stage IVA) with pleural dissemination. Initial staging was performed using contrast-enhanced computed tomography (CT) of the chest and abdomen, brain magnetic resonance imaging (MRI), and 18F-fluorodeoxyglucose positron emission tomography/computed tomography (18F-FDG PET/CT). No brain metastases were detected at diagnosis. Combination therapy with carboplatin, etoposide, and atezolizumab was initiated in January 202X-3. Since disease progression was observed during atezolizumab maintenance therapy, amrubicin was started as a second-line treatment in August 202X-3. Topotecan treatment was initiated in March 202X-1, followed by irinotecan treatment in June 202X-1. Contrast-enhanced CT demonstrated a 53-mm para-aortic lymph node lesion with heterogeneous attenuation and central low-density areas suggestive of necrosis in December 202X-1. In February 202X, a contrast-enhanced brain MRI revealed a new 9-mm right cerebellar metastasis with heterogeneous enhancement and no surrounding edema. No other measurable or non-target lesions were identified at that time. Palliative radiotherapy (30 Gy in 10 fractions) was administered to the para-aortic lymph node lesion in March 202X. Subsequently, stereotactic radiotherapy (32.5 Gy in five fractions) was delivered to the right cerebellar metastasis. Grade 1 radiation pneumonitis developed after radiotherapy to the para-aortic lymph node lesion and required no specific treatment. No radiation necrosis or other severe treatment-related adverse events were observed. Both irradiated lesions subsequently showed marked radiographic regression and were no longer measurable. At the initiation of tarlatamab in May 202X, no measurable target lesions or residual non-target lesions were identified on imaging. Given the sequential development of new lesions after multiple prior lines of systemic therapy and the concern for early systemic recurrence despite successful local treatment, tarlatamab was initiated as fifth-line therapy. At the initiation of tarlatamab, the patient was alert, afebrile, and hemodynamically stable, with normal oxygen saturation and no signs of respiratory distress. His ECOG PS was 0, and physical examination revealed no notable abnormalities. Tarlatamab was administered intravenously at 1 mg on day 1, followed by 10 mg on days 8 and 15. Thereafter, maintenance treatment with 10 mg was administered every two weeks, with occasional extension of the dosing interval to three weeks depending on the patient’s clinical condition or personal circumstances. During treatment, grade 2 cytokine release syndrome occurred following tarlatamab administration and resolved after treatment with an antipyretic and dexamethasone. After tarlatamab initiation, the previously irradiated lesions remained controlled, with no evidence of new disease until October 202X. However, in October 202X, a contrast-enhanced brain MRI revealed a new solitary 15-mm metastasis in the left cerebellar hemisphere. The lesion showed heterogeneous enhancement with partial ring enhancement without surrounding edema. Stereotactic radiotherapy (32.5 Gy in five fractions) was administered for this new brain metastasis, resulting in marked regression of the lesion without further systemic disease progression (Figure 2). Radiotherapy was delivered between tarlatamab administrations without interruption of treatment. No acute treatment-related adverse events were observed, and no late adverse events, including radiation necrosis, were identified during the available follow-up period. The patient remained neurologically asymptomatic, with no deterioration in PS or activities of daily living during follow-up. At the data cutoff, the patient remained alive without further disease progression for nine months after stereotactic radiotherapy.

Case 2

**Figure 3:**
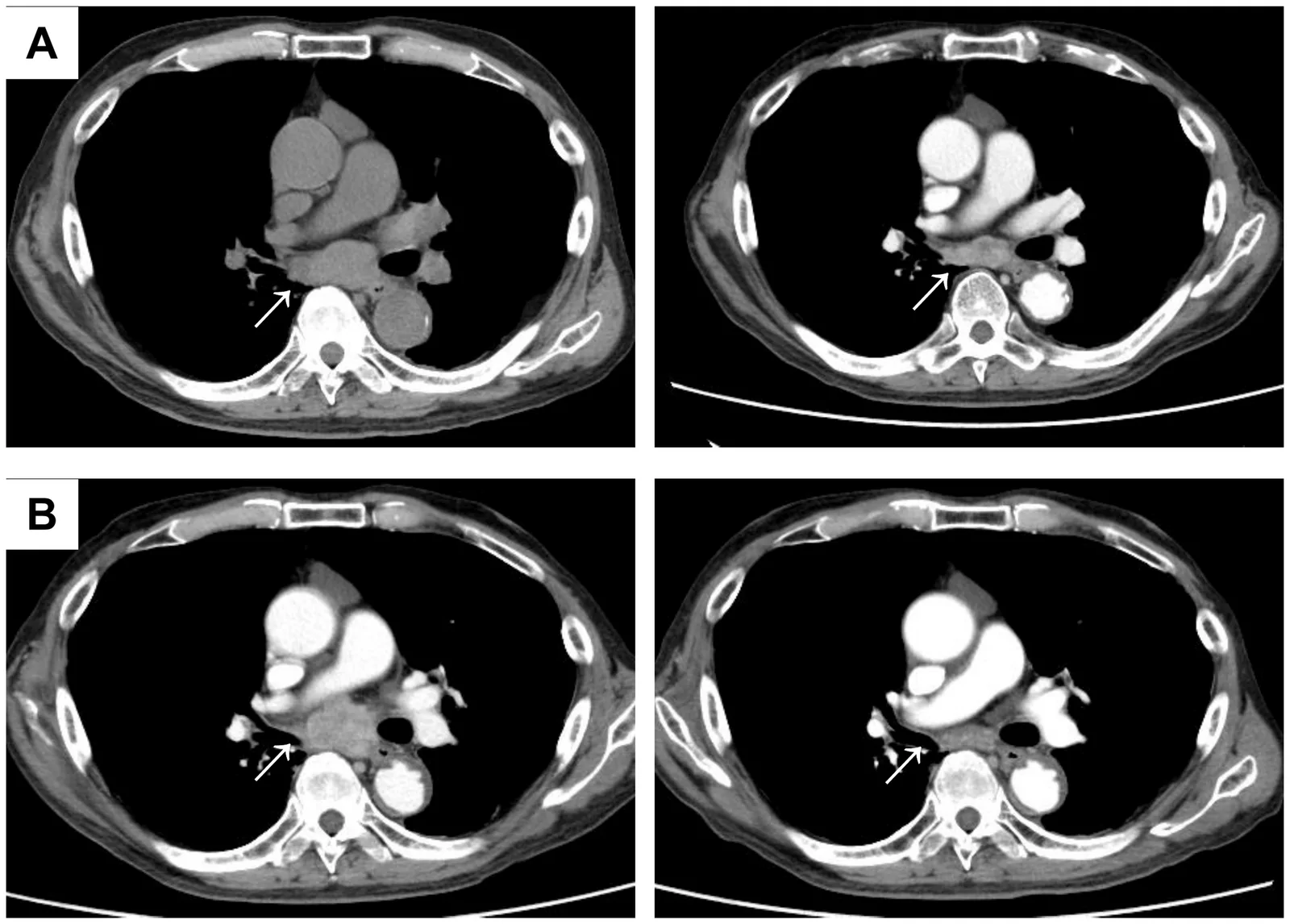
Radiological course of Case 2. (A) Computed tomography images of the subcarinal lymph node lesion before tarlatamab initiation (left) and after the initial tumor response (right). (B) Computed tomography images showing enlargement of the subcarinal lymph node lesion during tarlatamab treatment (left) and tumor reduction after hyperfractionated radiotherapy (right). White arrows indicate the subcarinal lymph node lesion.

**Figure 4:**
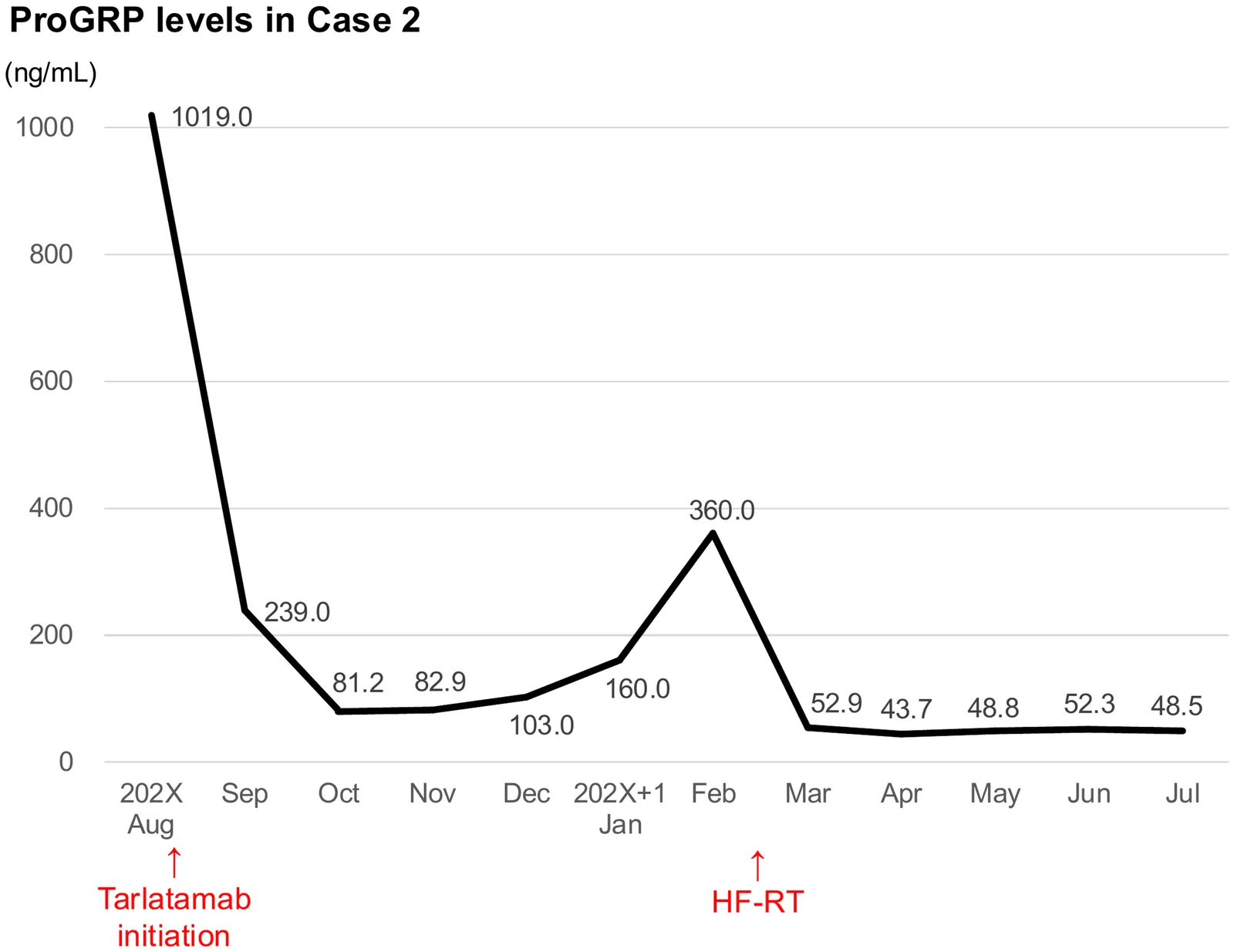
Changes in tumor marker levels during the clinical course. In Case 2, ProGRP levels closely reflected disease progression and response to radiotherapy. HF-RT: hyperfractionated radiotherapy; ProGRP: pro-gastrin-releasing peptide

A 78-year-old man with chronic obstructive pulmonary disease and a history of surgically treated squamous cell carcinoma of the left auricle was referred to our hospital after an abnormal chest shadow was detected on non-contrast CT in April 202X-2. Because endoscopic diagnosis was unsuccessful, a surgical lymph node biopsy was performed in July 202X-2, and SCLC was pathologically diagnosed. He had a 40-pack-year smoking history and an ECOG PS of 1 at diagnosis. He was diagnosed with ES-SCLC (cT4N2bM1a, stage IVA). Initial staging was performed using contrast-enhanced CT of the chest and abdomen, brain MRI, and 18F-FDG PET/CT. Pleural dissemination was identified as the basis for the M1a classification. Treatment with carboplatin plus etoposide was initiated in August 202X-2, and four cycles were completed by November 202X-2. An immune checkpoint inhibitor was not added because of severe pulmonary emphysema. After four cycles, the primary lung lesion, pleural dissemination, and metastatic lymph nodes had completely regressed, resulting in a complete radiographic response. As the planned four cycles had been completed and a complete response had been achieved, no additional chemotherapy was administered. Consolidative thoracic radiotherapy was not performed because no residual thoracic disease was visible on imaging, and the patient had severe pulmonary emphysema, raising concern about radiation-related pulmonary toxicity. As recurrence of multiple mediastinal lymph node metastases was detected on 18F-FDG PET/CT, platinum rechallenge with carboplatin plus etoposide was administered in December 202X-1. Although tumor regrowth was observed thereafter, amrubicin and irinotecan, which are associated with the risk of pulmonary toxicity, were avoided because of severe emphysema in the patient. Topotecan was unavailable because of a drug shortage at the time. In addition, paclitaxel was considered difficult to administer because of the patient's alcohol intolerance, as the formulation contained alcohol as a solvent. Therefore, off-label nab-paclitaxel was initiated in June 202X [5,6]. However, because disease progression was observed, tarlatamab was initiated as fourth-line therapy in August 202X. At the initiation of tarlatamab, the patient was alert and clinically stable, with normal vital signs, including oxygen saturation, and no respiratory distress. His ECOG PS was 1, and physical examination revealed no notable abnormalities. Tarlatamab was administered intravenously at 1 mg on day 1, followed by 10 mg on days 8 and 15. Thereafter, maintenance treatment with 10 mg was administered every three weeks because frequent hospital visits were difficult owing to his advanced age and long travel distance. Grade 2 cytokine release syndrome developed during the initial phase of tarlatamab treatment and resolved with antipyretic therapy and dexamethasone. On a non-contrast CT performed in July 202X, the subcarinal lymph node measured 25 mm before the initiation of tarlatamab. On a subsequent contrast-enhanced CT performed in October 202X, the lymph node had temporarily decreased to 17 mm but continued to show heterogeneous enhancement, and the response was assessed as a partial response. However, the lymph node subsequently enlarged to 29 mm with heterogeneous enhancement in February 202X+1, and the patient was diagnosed with oligoprogressive disease limited to the subcarinal lymph node lesion. The serum pro-gastrin-releasing peptide (ProGRP) levels, which initially decreased after the initiation of tarlatamab treatment, increased again in association with lymph node regrowth. Given that the progression was limited to the subcarinal lymph node lesion, a decision was made to proceed with definitive-dose radiotherapy. To minimize the interruption of the tarlatamab dosing schedule, hyperfractionated radiotherapy (45 Gy in 30 fractions over three weeks) was initiated in March 202X+1 [7]. The gross tumor volume was defined as the radiographically visible subcarinal lymph node lesion on treatment-planning CT. The clinical target volume was created by adding a 5-mm margin to the gross tumor volume, and the planning target volume was created by adding a further 5-mm margin to the clinical target volume. No elective nodal irradiation was performed, and no other lymph node stations were included in the irradiated field. Radiotherapy was delivered between tarlatamab administrations, and one scheduled dose was postponed by one week during radiotherapy. Following hyperfractionated radiotherapy, grade 1 radiation dermatitis developed and resolved without specific treatment. No other clinically significant radiotherapy-related adverse events, including radiation pneumonitis, were observed during follow-up. Following the completion of radiotherapy, ProGRP levels rapidly normalized, and contrast-enhanced CT demonstrated marked shrinkage of the lymph node lesion (Figures 3, 4). At the data cutoff, the patient remained alive, and the subcarinal lymph node lesion remained controlled for four months after hyperfractionated radiotherapy.

In both cases, disease control remained favorable after radiotherapy. Given that ES-SCLC is generally sensitive to radiotherapy and chemotherapy but is characterized by rapid disease progression and limited treatment options [4], tarlatamab treatment was continued after discussion with both patients, with the aim of maintaining disease control and preserving subsequent treatment options.

## Discussion

In Case 1, a new solitary brain metastasis developed despite sustained extracranial disease control. Previous reports have described rapid intracranial responses to tarlatamab in patients with untreated brain metastases [8]. However, a recent case report demonstrated discordant responses, with extracranial tumor regression despite intracranial disease progression during tarlatamab treatment [9]. Similarly, our Case 1 showed discordant intracranial and extracranial disease control. These findings suggest that intracranial and extracranial responses to tarlatamab may differ in some patients and that continued brain MRI surveillance may be warranted even when extracranial disease remains controlled. In Case 2, progression was limited to regrowth of the subcarinal lymph node lesion, with no evidence of progression at other sites. Since systemic disease was generally controlled during tarlatamab treatment and progression was confined to a single lesion in both cases, these cases represented oligoprogression. In both cases, tarlatamab had been introduced as a later-line treatment; therefore, a change in systemic therapy would have left very limited subsequent treatment options. For patients with oligoprogression, a strategy of continuing effective systemic therapy while applying local treatment to progressive lesions has been increasingly reported, particularly for advanced non-small cell lung cancer (NSCLC) [10,11]. A phase two trial comparing local consolidative therapy with maintenance therapy or observation demonstrated significant improvements in PFS [10]. Although local treatment strategies for oligoprogression are being established for NSCLC, therapeutic strategies for oligoprogression in ES-SCLC remain insufficiently defined. Notably, the 12-month PFS rate in the tarlatamab group of the DeLLphi-304 trial was 20% [2], suggesting that durable disease control can be achieved in a subset of patients. Therefore, a treatment strategy that enables the continuation of tarlatamab through the addition of local therapy may contribute to prolonged disease control and be clinically meaningful. In the present cases, radiotherapy to the progressing site was administered while continuing tarlatamab treatment, drawing on treatment strategies established for NSCLC in which local therapy is used to prolong the benefit of effective systemic treatment [10]. Consequently, the systemic disease remained generally controlled, and tarlatamab treatment was continued in both cases. A recent multi-institutional study of patients with SCLC who received concurrent radiotherapy and tarlatamab reported a low incidence of severe toxicity, frequent local tumor responses, and a trend toward improved overall survival in patients who received radiotherapy [12]. Consistent with these findings, radiotherapy was completed in both of our patients without clinically significant toxicity requiring discontinuation of tarlatamab; no radiotherapy-related adverse events occurred in Case 1, and only grade 1 radiation dermatitis was observed in Case 2. Whereas the previous study primarily evaluated the overall feasibility and outcomes of concurrent treatment [12], our cases specifically illustrate that radiotherapy to isolated oligoprogressive lesions may permit continuation of otherwise effective tarlatamab therapy.

A prospective phase two trial in advanced NSCLC reported that among patients who received radiotherapy for lesions that progressed during immune checkpoint inhibitor therapy, 93.8% were able to continue immune checkpoint inhibitor treatment, and the disease control rate at three months was 62.5% [13]. These findings suggest that radiotherapy may be a useful strategy for maintaining ongoing systemic therapies, including immunotherapy. Although the histology and therapeutic agents differ, radiotherapy in these two patients with ES-SCLC may have enabled continued treatment with tarlatamab and contributed to sustained disease control.

This report has several limitations. First, it describes only two cases. Second, it remains unclear which patients are most likely to benefit from a treatment strategy that combines tarlatamab with radiotherapy. Third, the follow-up period after radiotherapy was limited, particularly with regard to the assessment of late radiotherapy-related toxicity and long-term disease control. Further studies are required to evaluate the efficacy and safety of combining tarlatamab with radiotherapy.

## Conclusions

Radiotherapy may enable the continuation of tarlatamab treatment in patients with ES-SCLC who develop oligoprogression during therapy. These two cases provide preliminary evidence that radiotherapy to sites of oligoprogression may allow continuation of tarlatamab treatment in selected patients; however, the limited follow-up period precludes definitive conclusions. In both cases, radiotherapy was completed without treatment-related toxicity requiring interruption of tarlatamab. In selected patients with otherwise controlled systemic disease, continuation of effective systemic therapy may help preserve disease control and subsequent treatment options.
